# Identification of the course of plastic stent‐induced pancreatic duct mucosal change in chronic pancreatitis using peroral pancreatoscopy (with video)

**DOI:** 10.1002/deo2.70050

**Published:** 2025-01-13

**Authors:** Kensuke Takuma, Naoki Okano, Yusuke Kimura, Koji Watanabe, Hiroki Nakagawa, Kensuke Hoshi, Masashi Miura, Naobumi Tochigi, Yoshinori Igarashi, Takahisa Matsuda

**Affiliations:** ^1^ Division of Gastroenterology and Hepatology Toho University Omori Medical Center Tokyo Japan; ^2^ Department of Surgical Pathology Toho University Omori Medical Center Tokyo Japan

**Keywords:** chronic pancreatitis, endoscopic pancreatic stenting, main pancreatic duct stricture, peroral pancreatoscopy, stent‐induced ductal change

## Abstract

Stent‐induced ductal change is a complication of endoscopic treatment of the main pancreatic duct in chronic pancreatitis. Most previous reports have been based on morphological duct changes observed via pancreatography. Here, we describe a case of stent‐induced ductal change in which the course of the mucosal changes was observed through peroral pancreatoscopy with a videoscopy.

The patient presented with chronic alcoholic pancreatitis. Main pancreatic duct stenosis in the pancreatic head was identified and a 10‐Fr plastic stent was inserted. Follow‐up pancreatography revealed a focally elevated duct change of approximately 4.5 mm at the distal tip of the stent, and peroral pancreatoscopy was performed. The elevated ductal change was identified as a clear villiform‐like nodular mucosal change with the spread of pale papillary and granular mucosa. Reassessment after stent removal showed an improvement in the elevated mucosal lesion, with residual discoloration and mucosal retraction suggestive of scarring. The stent may cause irreversible changes, undetectable morphologically by pancreatography, likely underestimating stent‐induced ductal change in chronic pancreatitis.

## 1 INTRODUCTION

Endoscopic pancreatic duct stenting has been shown to be an effective technique for the treatment of symptomatic chronic pancreatitis (CP) with main pancreatic duct (MPD) stricture.^1^ However, stent‐induced ductal changes (SIDC) have been reported as a complication of this procedure.^1^, ^2^ SIDC should be considered a serious complication that potentially affects the long‐term outcomes of patients with CP. However, few studies have evaluated SIDC in CP, with most involving morphological assessments using endoscopic retrograde pancreatography (ERP). To our knowledge, no reports have assessed the mucosal or tissue changes in SIDC under direct vision. Here, we report the course of mucosal changes in CP with MPD stricture caused by endoscopic pancreatic duct stenting observed via peroral pancreatoscopy (POPS) with a videoscopy (Video S1).

### 1.1 Case report

A 68‐year‐old man with a history of acute alcoholic pancreatitis reported to our department. The patient had a history of chronic alcohol consumption and presented with persistent abdominal pain. Abdominal computed tomography revealed a pancreatic calculus of approximately 13 mm in the pancreatic head and MPD dilatation of the caudal pancreas. Based on the Japanese clinical diagnostic criteria,^3^ the patient was diagnosed with definite CP. The pain was thought to be caused by increased pressure within the pancreatic ductal system from impaired MPD outflow, leading to a referral for endoscopic treatment.

**FIGURE 1:**
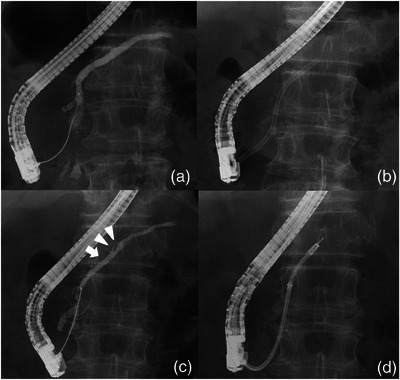
Pancreatogram before the placement of 10‐Fr S‐type plastic stent shows no obvious focal elevated ductal changes (a). The 10‐Fr S‐type plastic stent placed in the main pancreatic duct for chronic pancreatitis treatment (b). Pancreatogram at the time of stent removal showing a focal elevated duct change (arrowhead) at the distal end of the stent, and no significant changes (arrow) at the flat elevated mucosal area (c). Peroral pancreatoscope was inserted into the main pancreatic duct to assess the mucosal change at the stent tip. The lesion was biopsied under direct vision using forceps (d).

**FIGURE 2:**
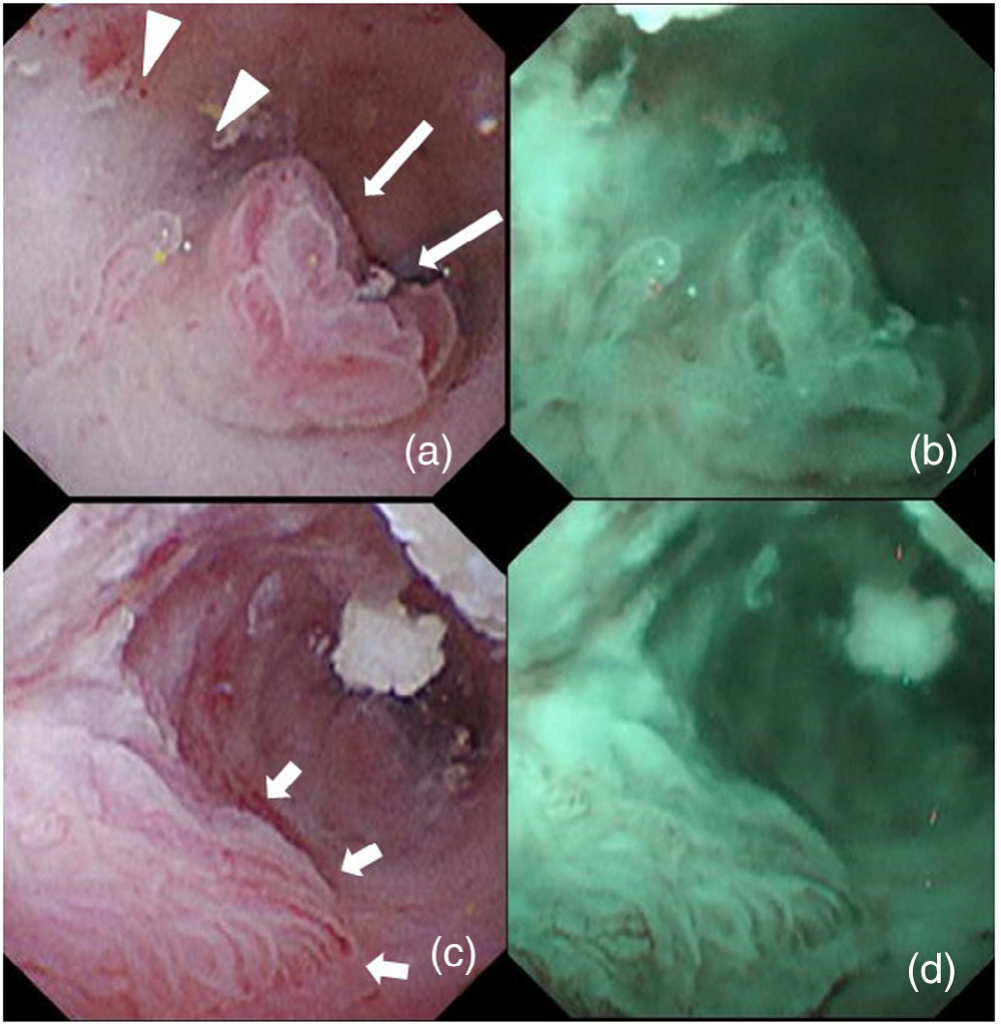
Peroral pancreatoscopy images showing villiform‐like nodular mucosal change (long arrow) at the distal end of the stent. Surrounding areas display pale papillary and granular mucosa (arrowhead), indicating inflammatory changes (a, b). White‐light imaging and narrow‐band imaging of the flat elevated mucosal area (short arrow). Narrow‐band imaging does not show obvious irregular blood vessels; however, dilated and tortuous vessels are seen, suggesting mucosal congestion and edema in the proximal pancreatic duct (c, d).

**FIGURE 3:**
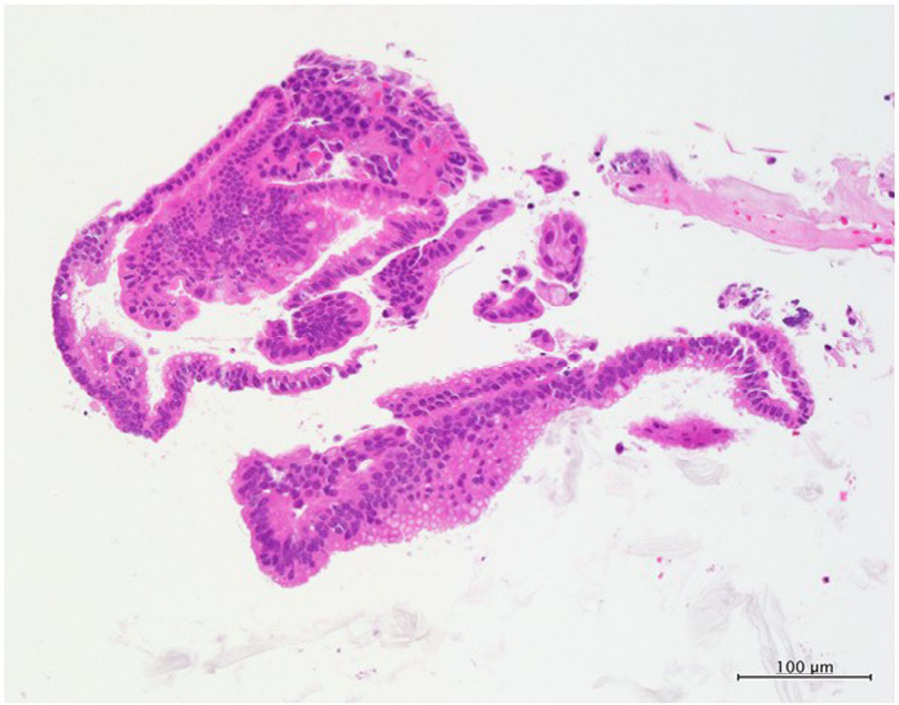
Histological analysis of the villiform‐like nodular mucosal change in the main pancreatic duct. The biopsy specimen shows inflammatory exudate and glandular epithelium with mildly enlarged cell nuclei, confirming reactive changes without evidence of neoplasia.

At our hospital, ERP revealed impacted pancreatic headstones. We performed endoscopic pancreatic sphincterotomy and placed a 5‐Fr endoscopic nasopancreatic drainage tube in the MPD. Extracorporeal shock wave lithotripsy was performed in 10 sessions (20,000 shots), and most stones were removed via subsequent endoscopic procedures. MPD stenosis in the pancreatic head was identified and a 10‐Fr/6 cm S‐type plastic stent (PS) was placed to achieve a dilating effect (Olympus, Tokyo, Japan). After 104 days, we performed a follow‐up ERP to evaluate MPD strictures and residual pancreatic stones. Pancreatography revealed a focal elevated duct change of approximately 4.5 mm at the distal tip of the PS (Figure 1a–c). We attempted to evaluate the pancreatic duct changes using POPS. POPS was performed using the mother‐baby scope method with saline injection. The mother scope was TJF‐260 V (Olympus), and the baby scope was CHF‐B290 (Olympus). The observation device used was the EVIS X1 endoscopy system (Olympus). We successfully inserted the baby scope into the MPD (Figure 1d). Details of the elevated mucosal changes detected by pancreatography were observed using POPS. An elevated ductal change at the tip of the stent was identified as a clear villiform‐like nodular mucosal change. In addition, the spread of the pale papillary and granular mucosa around the villiform mucosa was confirmed. Narrow‐band imaging did not reveal obvious irregular blood vessels (Figure 2a,b). The pancreatic duct mucosa proximal to the elevated lesion showed erosion, and flat elevated mucosal changes were confirmed, which were not detected morphologically on ERP (Figure 1c). Dilated and tortuous blood vessels were observed, suggesting that the congested blood vessels were associated with edematous changes in the mucosa (Figure 2c,d). Biopsy of the villiform mucosal lesion and flat elevated mucosal changes was performed using Endojaw slim forceps (Olympus) for histological diagnosis. Histological findings from the two biopsy sites revealed reactive mucosal changes but no neoplastic alterations (Figure 3). The identified mucosal changes were strongly suspected to be caused by SIDC; therefore, the PS was removed.

**FIGURE 4:**
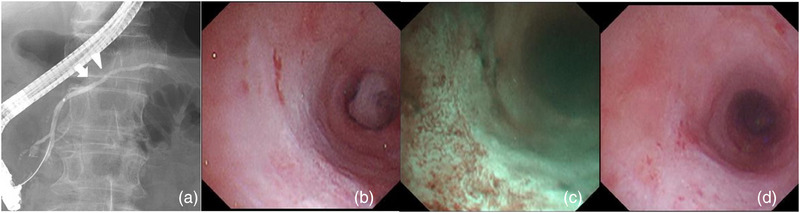
Pancreatogram 95 days after the removal of the 10‐Fr S‐type plastic stent. The previously identified locations are marked: the arrowhead indicates the villiform‐like nodular mucosal change, and the arrow indicates the flat elevated mucosal change. The previously identified focal ductal change has improved (a). Peroral pancreatoscopy showing the site of the previously identified villiform‐like nodular mucosal change under white‐light imaging and narrow‐band imaging. The mucosa appears discolored and retracted, without visible vessels, consistent with scarring (b, c). A flat elevated mucosal area has also shown retraction and discoloration, further indicating scarring at the site of the previous inflammatory change (d).

After 95 days, MPD mucosal changes were re‐evaluated endoscopically. The ERP findings revealed morphological improvement (Figure 4a). POPS was also performed to assess the progression of mucosal changes. No elevated mucosal lesions were identified. Mucosal discoloration and retraction without vascular findings were observed at the same site using white‐light imaging and narrow‐band imaging, suggesting scarring (Figure 4b–d). The elevated mucosal changes were attributed to the inflammatory changes caused by contact with the PS. Pancreatography revealed no dilation of the MPD after PS removal, and the POPS was successfully inserted. Consequently, the MPD stricture in the pancreatic head region was considered improved, and the PS was not reinserted. The patient has since been followed up as an outpatient for CP with no significant changes in symptoms.

## 2 DISCUSSION

The causes of SIDC are not yet fully understood. Previous reports have suggested that SIDC develops at the stent tip and improves over time following PS removal, implying that these changes may result from local edema or acute inflammation because of direct trauma to the ductal epithelium.^2^, ^4^, ^5^, ^6^ We reported the incidence of SIDC in 10‐Fr PS is as high as 31.1%, but therapeutic risk factors such as the choice of stent length and computed tomography findings of axial discrepancy between MPD and PS tip have not been identified.^2^ In this case, we observed SIDC under direct vision with POPS and identified villiform and papillary elevations along with erosive changes. Morphologically, the duct changes appeared to be reversible; however, scarring suggested permanent damage. This is the first report documenting a course on SIDC using POPS with videoscopy.

The development of imaging diagnosis using videoscopy has dramatically improved, leading to a wide range of clinical applications.^7^, ^8^ In this case, ERP recognized only the elevated lesion at the tip of the PS, but POPS confirmed more extensive mucosal changes along the PS. The MPD has a complex three‐dimensional course. Therefore, it may be difficult to recognize the overall image of the focal elevated changes. The histological findings at the two locations were similar; however, the differences in appearance under direct visualization are likely attributed to mucosal damage caused by scratching from the flap at the tip of the PS and irritation from contact with the stent body. SIDCs are defined as both elevated and stricture changes.^2^ In this case, the villiform mucosal change at the tip of the stent was recognized as an elevated type. The pale papillary and granular mucosa surrounding the villiform mucosa was thought to represent inflammation spreading to the adjacent areas. Continued PS placement for elevated‐type SIDC can result in more pronounced stricture changes.^2^ Persistence of inflammation caused by PS contact may lead to mucosal changes, such as growth or extension. Moreover, it is important to differentiate between the identified elevated MPD lesions and neoplastic lesions. In this case, narrow‐band imaging did not reveal any obvious irregular blood vessels. However, it is essential to differentiate the mucosal structure with villiform, papillary, and granular elevations from intraductal papillary mucinous neoplasms (IPMN). IPMN are characterized by various papillary growths of mucus‐producing neoplastic epithelium and comprises a spectrum of epithelial changes ranging from hyperplasia to carcinoma.^8^, ^9^ In addition to the pathological diagnosis by direct visual biopsy, follow‐up evaluation after elimination of the cause of SIDC is important to distinguish it from neoplastic lesions. In this case, evaluation of the mucosal course using POPS was particularly useful.

Previous reports have shown that residual SIDC after PS removal, as assessed morphologically by ERP, is often determined to be an irreversible ductal change.^10^ Direct observation with follow‐up POPS also demonstrated the disappearance of the elevated lesions. However, mucosal findings showed discoloration and retraction of the mucosa, suggesting scarring, which was suspected to be an irreversible change. Sherman et al.^5^ reported that endoscopic ultrasound evaluation after morphological pancreatic duct improvement detected persistent local parenchymal changes, similar to those in CP, over a long period of time. PS may cause irreversible mucosal and parenchymal changes that cannot be noted morphologically by ERP, thus likely underestimating stent‐induced changes in CP. It is important to emphasize that the treatment of PS requires attention to the potential pancreatic damage in the non‐stenotic areas.

This report is the first to demonstrate that POPS with videoscopy is effective in identifying the course of SIDC. In recent years, remarkable progress has been made regarding the use of baby scopes for better performance of endoscopic retrograde cholangiopancreatography. In the future, the role of POPS in the endoscopic treatment of CP is likely to become even more significant.

## CONFLICT OF INTEREST STATEMENT

None.

## PATIENT CONSENT STATEMENT

Informed consent for publication was obtained from the patient.

## Supporting information



1. Pancreatography at plastic stent removal shows the focal elevated change of the main pancreatic duct at the distal end of the stent.
2. Peroral pancreatoscopy successfully inserted into the main pancreatic duct.
3. Peroral pancreatoscopy shows villi‐like nodular mucosal change with a spread of pale papillary granular mucosa at the point indicated by pancreatography.
4. Erosion and flat elevated mucosal change are seen in the proximal pancreatic duct.
5. A biopsy of the villiform mucosal lesion is performed using forceps.
6. Pancreatography at 95 days after removal of the plastic stent shows no obvious changes in the main pancreatic duct.
7. The elevated mucosal lesions cannot be visualized with peroral pancreatoscopy; however, discoloration and retraction of the mucosa are observed, suggesting scarring.

The course of the mucosal changes in the main pancreatic duct because of endoscopic pancreatic duct stenting observed via peroral pancreatoscopy with videoscopy.
