# Supplementary material for: Identification of the course of plastic stent‐induced pancreatic duct mucosal change in chronic pancreatitis using peroral pancreatoscopy (with video)
Source: DEN Open. 2025 Jan 13;5(1):e70050. doi: 10.1002/deo2.70050 (PMC11727273; doi:10.1002/deo2.70050)
Supplement: Supplementary file 1 — 1. Pancreatography at plastic stent removal shows the focal elevated change of the main pancreatic duct at the distal end of the stent. 2. Peroral pancreatoscopy successfully inserted into the main pancreatic duct. 3. Peroral pancreatoscopy shows villi‐like nodular mucosal change with a spread of pale papillary granular mucosa at the point indicated by pancreatography. 4. Erosion and flat elevated mucosal change are seen in the proximal pancreatic duct. 5. A biopsy of the villiform mucosal lesion is performed using forceps. 6. Pancreatography at 95 days after removal of the plastic stent shows no obvious changes in the main pancreatic duct. 7. The elevated mucosal lesions cannot be visualized with peroral pancreatoscopy; however, discoloration and retraction of the mucosa are observed, suggesting scarring. [file DEO2-5-e70050-s001.docx]

**Video　text**

1. Pancreatography at plastic stent removal shows the focal elevated change of the main pancreatic duct at the distal end of the stent.

2. Peroral pancreatoscopy successfully inserted into main pancreatic duct.

3. Peroral pancreatoscopy shows villi-like nodular mucosal change with spread of pale papillary granular mucosa at the point indicated by pancreatography.

4. Erosion and flat elevated mucosal change are seen in the proximal pancreatic duct.

5. Biopsy of the villiform mucosal lesion is performed using forceps.

6. Pancreatography at 95 days after removal of the plastic stent shows no obvious changes in the main pancreatic duct.

7. The elevated mucosal lesions cannot be visualized with peroral pancreatoscopy; however, discoloration and retraction of the mucosa are observed, suggesting scarring.
